# Complete Genome Sequence of Bluetongue Virus Serotype 8, Which Reemerged in France in August 2015

**DOI:** 10.1128/genomeA.00163-16

**Published:** 2016-04-14

**Authors:** Emmanuel Bréard, Corinne Sailleau, Hélène Quenault, Pierrick Lucas, Cyril Viarouge, Fabrice Touzain, Aurore Fablet, Damien Vitour, Houssam Attoui, Stéphan Zientara, Yannick Blanchard

**Affiliations:** a Université Paris-Est ANSES Alfort, UMR 1161 ANSES/INRA/ENVA, Maisons-Alfort, France; b Anses, Laboratory of Ploufragan, Unit of Viral Genetics and Biosafety, Ploufragan, France

## Abstract

We announce here the complete genome sequence (coding and noncoding) of the bluetongue virus (BTV) serotype 8, isolated from a ram in Allier department, France, 2015. Sequence analysis confirms the reemergence of the BTV-8 strain that previously circulated in France until 2009 and other European countries until 2010.

## GENOME ANNOUNCEMENT

Bluetongue is an infectious, noncontagious, and arthropod-borne viral disease of domesticated and wild ruminants (1) caused by bluetongue virus (BTV) (*Orbivirus* genus, *Reoviridae* family). The BTV genome consists of 10 segments of double-stranded RNA. To date, a total of 27 distinct BTV serotypes (BTV-1 to BTV-27) have been identified (2–4).

In August 2015, BTV-8 was detected in a sick ram showing clinical signs of BTV infection in central France (department of Allier) (5). Since its first detection, the virus continued spreading through autumn until the end of December. Sixteen French departments were affected with a total 150 infected herds, of which 95 herds (63.3%) were located in neighboring Allier and Puy-de-Dôme departments (identified as the epicenter of the current BTV-8 outbreak).

Although the origin of the BTV-8 responsible for the 2006 outbreak has never been defined, the BTV-8 strain circulating in France in 2015 has an almost identical sequence to that of the 2008 French isolates. It is likely that the current BTV-8 strain has been circulating, with a low prevalence in the field, possibly by infecting domestic and/or wild ruminants, since it was last detected in France in 2009.

The BTV-8 strain, designated BTV8-15-01, was isolated from blood after a single passage on BSR cells (5). Next-generation sequencing was performed on RNA extracts from infected cultures. RNA extraction was performed using TRIzol LS reagent (Life Technologies, Germany), as described by the manufacturer. Trimmomatic and bowtie2 were used for cleaning and aligning reads (6), and SAMtools was used to create a consensus sequence (7). Alignment was realized using progressiveMauve (8). Sequencing permitted the determination of the full-length genome. Sequence analysis of the 10 double-stranded RNA segments (Seg) that constitute the BTV genome showed a close relationship to BTV-8 isolated in France in 2008, which was fully sequenced at the Pirbright Institute (9) (the strains are identified in GenBank as bluetongue virus 8 isolates FRA2008/28 and FRA2008/29). The sequences of the noncoding regions in Seg-1 to Seg-10 were 100% identical to those of BTV8 FRA2008/28 and FRA2008/29. The encoded proteins had sizes in agreement with those known for BTV-8 isolates available in GenBank. Nineteen substitutions were identified in 18,444 coding nucleotides, with a consequence of 4 amino acid changes (1 change in each of Seg-1, 3, 8, and 9). Indeed, these results clearly show that the BTV-8 isolated in 2015 is identical to the BTV-8 strain that circulated in France and in Europe until 2010. These results also seem to exclude the hypothesis of a new introduction.

### Nucleotide sequence accession numbers.

The complete coding genome sequences of the novel BTV-8 have been deposited in GenBank under accession numbers KU569990 to KU569999. The version described in this paper is the first version.
